# Supplementary material for: Kinematic analysis of the underwater glide phase during the push-off in competitive swimmers
Source: PLoS One. 2024 Jan 2;19(1):e0296101. doi: 10.1371/journal.pone.0296101 (PMC10760865; doi:10.1371/journal.pone.0296101)
Supplement: Attachment — Submitted filename: Comments Reviewer 2.docx [file pone.0296101.s002.docx]

Reviewer #2:

- I read this work with interest and in my opinion authors did a good article. Some deficiencies are given under these lines, but an extensive review is unnecessary.

*Thank you for your kind words. I have tried to improve the manuscript according to your suggestions. However, I would be grateful for any further advice.*

- INTRODUCTION

Well written, but too little introduction to the topic and too much discussion and scientific examples. Much part of the text is suitable for discussion. Please add some basic information about underwater swimming, glides, breakouts, differences in glides/strokes etc.

*Thank you for your comments. Significant changes have been made to this section. Literature on underwater swimming and gliding has been added. In addition, the scientific examples have been removed and more state of the art has been included.*

- MATERIAL AND METHODS

L77-78 : Consistently one or two digits after the decimal point. I advise one.

*Corrected.*

L86-87 : Add some information about season period (kilometers, intensity, hours etc.)

*Thanks for your comment. The "Participants" section has been modified with some advice from the other reviewer. I would be grateful if you could review it and give me feedback.*

- DESIGN AND PROCEDURES

L104-105 : If breaststroke was not in research, give some information in introduction that this stroke and glide is much different from others.

*Thank you for your comment. This information has been added to the design and procedure section, explaining why the underwater breaststroke glide was discarded.*

*“The breaststroke was not filmed during the race because the swimmers had to finish the glide phase with the dolphin kick”.*

L111-114 : Please use one term – velocity or speed. Add SI units to all.

*Corrected.*

- RESULTS

L136 : Table 2. Add SI Units in first column (also Table 3). The female and male were exactly the same results in time [s] ?

*Corrected. Suggestions for units in Tables 2 and 3 have been added. So it is correct that both sexes got the same values for slippage. This is described in the discussion.*

- DISCUSION

The discussion is well written, but with fragments that are not suitable for publication because the number of results is too detailed. Less details and more typical conclusions please. I strongly recommend that you should use a typical thesis conclusion and compare it with other researchers. Especially in conclusion section, You should not use exact results, numbers and units etc. – only fruits of Your labour.

*Thank you for your recommendations. I have proceeded to a more complete restructuring, less detailed numerically and more comparative. The exact results, numbers and units were then removed from the conclusion section.*
